# Supplementary figures and images for: Carbon Monoxide (CO) as a Retinal Regulator of Heme Oxygenases -1, and -2 (HO’s) Expression
Source: Biomedicines. 2022 Feb 1;10(2):358. doi: 10.3390/biomedicines10020358 (PMC8962416; doi:10.3390/biomedicines10020358)

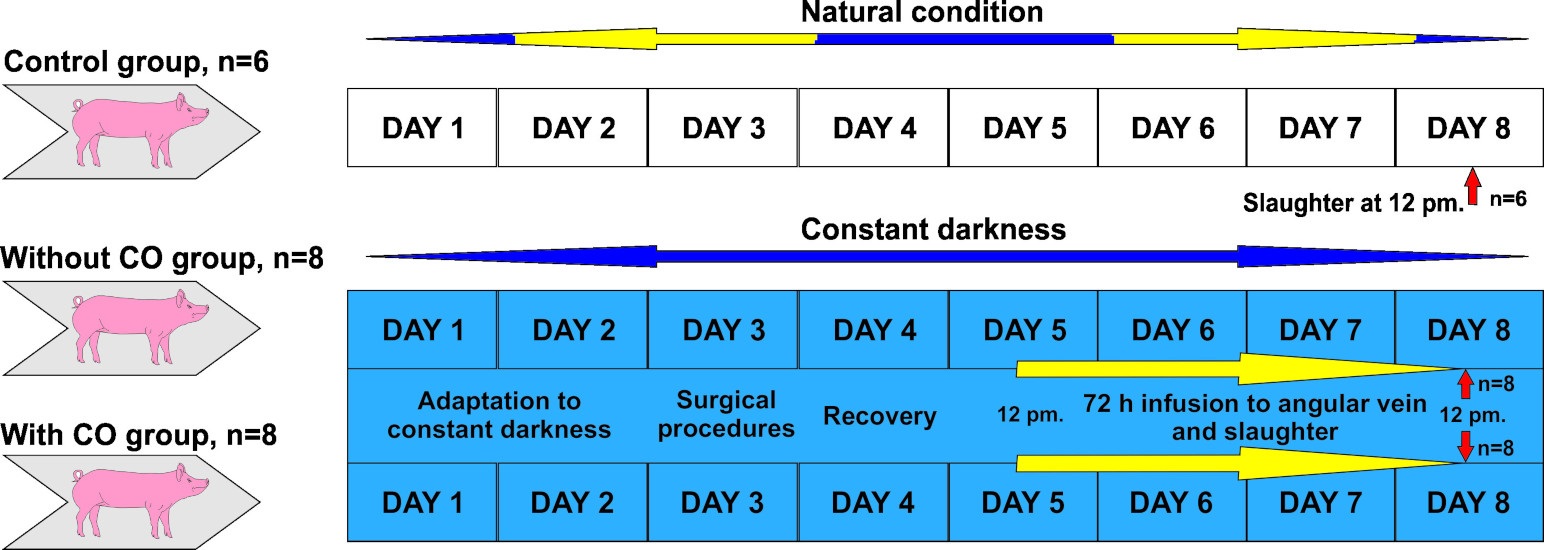

Supplement: Supplementary file 1 [file biomedicines-10-00358-s001.zip › biomedicines-1567785-supplementary.jpg]
